# Exploring the Transient Microbe Population on Citrus Butterfly Wings

**DOI:** 10.1128/spectrum.02055-21

**Published:** 2022-07-20

**Authors:** P. D. Kamala Jayanthi, Meenal Vyas

**Affiliations:** a Division of Crop Protection, ICAR- Indian Institute of Horticultural Research, Bengaluru, Karnataka, India; University of Minnesota

**Keywords:** citrus butterfly, entomopathogenic, symbionts, wing microbes

## Abstract

Microbes carve out dwelling niches in unusual environments. Insects, in general, have been hosts to microbes in different ways. Some insects incorporate microbes as endosymbionts that help with metabolic functions, while some vector pathogenic microbes that cause serious plant and animal diseases, including humans. Microbes isolated from insect sources have been beneficial and a huge information repository. The fascinating and evolutionarily successful insect community has survived mass extinctions as a result of their unique biological traits. Wings have been one of the most important factors contributing to the evolutionary success of insects. In the current study, wings of Papilio polytes, a citrus butterfly, were investigated for the presence of ecologically significant microbes within hours of eclosing under aseptic conditions. Scanning electron microscopy (SEM) revealed the presence of bacteria dwelling in crevices created by a specific arrangement of scales on the butterfly wing. A total of 38 bacterial isolates were obtained from the patched wings of the citrus butterfly, and *Bacillus* spp. were predominant among them. We probed the occurrence of these microbes to assess their significance to the insect. Many of the isolates displayed antibacterial, antifungal, and biosurfactant properties. Interestingly, one of the isolates displayed entomopathogenic potential toward the notorious agricultural pest mealybug. All the wing isolates were seen to cluster together consistently in a phylogenetic analysis, except for one isolate of Bacillus zhangzhouensis (Papilio polytes isolate [Pp] no. 28), suggesting they are distinct strains.

**IMPORTANCE** This is a first study reporting the presence of culturable microbes on an unusual ecological niche such as butterfly wings. Our findings also establish that microbes inhabit these niches before the butterfly has contact with the environment. The findings in this report have opened up a new area of research which will not only help understand the microbiome of insect wings but might prove beneficial in other specialized studies.

## INTRODUCTION

As a daunting community with thousands of species, insects are among the most evolutionarily successful organisms that have evolved to inhabit various ecological niches (1). As providers of several advantages to insects with respect to mobility, mate selection, escape from predators, and host expansion, wings have been one of the primary reasons for the evolutionary success of insects (2, 3). Human fascination and curiosity toward insect wings has inspired several improvisations in technological designs (4, 5). The stunning hues on butterfly wings play crucial roles in their survival, such as attracting mates (6, 7), exhibiting mimicry, and escaping from predators (8, 9).

Surface and structural studies of insect wings have suggested them to be among the most hydrophobic surfaces in nature. Up close, the arrangement of scales on a butterfly wing forms microscopic crevices that provide an excellent niche for microbes to thrive. They have the potential to either develop into symbiotic associations or exist as transient populations. The latter might be a more possible outcome when associations form on the exoskeleton. Insects have established symbiotic relationships with microbes to be evolutionarily fit in various aspects. Some bark beetle wings are known to harbor multiple symbiotic associations from different kingdoms (10). Microbes exist as endosymbionts in insect guts, helping either in digestion or synthesis of essential nutrients such as amino acids (11). Microbes have been shown to play a role in the maturation of reproductive organs and also drive sex bias in progeny, such as in the case of *Wolbachia* (12, 13). In some insects, such as Acraea encedon (14), *Drosophila* (15), and Armadillidium vulgare (16), microbes have been reported to alter cuticular volatile cues for mate attraction, which are good examples of their exoskeleton associations. Occurrence of microbes on the exoskeleton has been recorded in some insects (17), but their function as stable colonies has been difficult to assess. As mentioned earlier, the arrangement of scales on butterfly wings provides a good thriving place for microbes which could be either beneficial or pathogenic to the insect. Exploration of microbial communities on butterfly wings has not been reported so far; hence, a preliminary study was carried out to assess the presence of microbes and understand their ecological significance. This report compiles and presents data that reveal the presence of bacteria with distinct characteristics on citrus butterfly wings. A few of the isolates identified could possibly have an application in biocontrol of specific insect pests. Our findings open up an unexplored world of wing microbes that might have important roles in nature. The results and downstream possibilities have been discussed further.

## RESULTS

### Isolation and identification of bacteria.

As a primary study exploring the existence of microbial communities on Papilio polytes wings, our group was keen to understand the significance of their presence on the insect wings. Clipped butterfly wings were therefore patched on nutrient media, and the colonies obtained were PCR amplified and sequenced for identification. A total of 38 bacterial isolates were obtained from 6 different patched wings of *P. polytes*, and the subsequent 16S rRNA sequencing identified the presence of Bacillus megaterium/Bacillus aryabhattai (*P. polytes* isolates [Pp] no. 1 to 4, 9 to 13, 15, and 16), Bacillus zhangzhouensis/Bacillus pumilus (Pp no. 5, 6, 17, 25, and 27 to 29), Bacillus safensis/*B. pumilus* (Pp no. 7, 18, 22, and 30), Bacillus wiedmannii/Bacillus proteolyticus (Pp no. 36), *Lysinibacillus* sp. (Pp no. 32), Lysinibacillus fusiformis (Pp no. 34, 35, and 37), Staphylococcus haemolyticus (Pp no. 8, 20, 21, 23, 24, 26, and 31), and Staphylococcus saprophyticus (Pp no. 33, 38, and 39) on butterfly wings (Table 1). *Bacillus* species were predominantly found among the isolates. Some Staphylococcus species were identified too, which are known to be opportunistic pathogens to humans. However, these were not studied further.

**TABLE 1:** Details of bacteria isolated from *Papilio polytes* wing surface

Isolate no.	Identified bacterium^*b*^	Accession no.	Functional properties^*a*^		
			AF	AB	BS
Pp no. 1	*B. aryabhattai*/B. megaterium (99.02)	MT733985	–	–	+
Pp no. 2	*B. aryabhattai*/B. megaterium (99.77)	MT733986	–	–	+
Pp no. 3	*B. aryabhattai*/B. megaterium (99.03)	MT733987	ND	–	–
Pp no. 4	*B. aryabhattai*/B. megaterium (99.02)	MT733988	ND	–	–
Pp no. 5	Bacillus zhangzhouensis/*B. pumilus* (99.15)	MT733989	ND	+	+
Pp no. 6	*B. zhangzhouensis*/*B. pumilus* (99.01)	MT733990	+	+	+
Pp no. 7	Bacillus safensis/*B. pumilus* (99.02)	MT733991	+	+	+
Pp no. 8	Staphylococcus haemolyticus (99.52)	MT733992	ND	ND	+
Pp no. 9	*B. aryabhattai*/B. megaterium (99.52)	MT733993	–	–	–
Pp no. 10	*B. aryabhattai*/B. megaterium (98.40)	MT733994	ND	–	–
Pp no. 11	*B. aryabhattai*/B. megaterium (96.64)	MT733995	–	–	+
Pp no. 12	*B. aryabhattai*/B. megaterium (98.91)	MT733996	ND	–	+
Pp no. 13	*B. aryabhattai*/B. megaterium (96.48)	MT733997	ND	–	+
Pp no. 15	*B. aryabhattai*/B. megaterium (99.46)	MT733998	ND	–	+
Pp no. 16	*B. aryabhattai*/B. megaterium (99.18)	MT733999	ND	–	–
Pp no. 17	*B. zhangzhouensis*/*B. pumilus* (98.55)	MT734000	+	+	+
Pp no. 18	Bacillus safensis/*B. pumilus* (98.92)	MT734001	+	+	+
Pp no. 20	*S. haemolyticus* (99.45)	MT734002	ND	ND	–
Pp no. 21	*S. haemolyticus* (97.32)	MT734003	ND	ND	–
Pp no. 22	*B. safensis*/*B. pumilus* (98.93)	ND	ND	+	–
Pp no. 23	*S. haemolyticus* (99.14)	MT734004	ND	ND	–
Pp no. 24	*S. haemolyticus* (99.07)	MT734005	ND	ND	+
Pp no. 25	*B. zhangzhouensis*/*B. pumilus* (99.34)	MT734006	+	+	+
Pp no. 26	*S. haemolyticus* (99.70)	MT734007	ND	NA	–
Pp no. 27	*B. zhangzhouensis*/*B. pumilus* (99.20)	MT734008	+	+	+
Pp no. 28	*B. zhangzhouensis*/*B. pumilus (*99.70)	MT734009	+	+	+
Pp no. 29	*B. zhangzhouensis*/*B. pumilus (*99.66)	MT734010	ND	+	+
Pp no. 30	*B. safensis*/*B. pumilus (*99.64)	MT734011	ND	+	+
Pp no. 31	*S. haemolyticus* (99.45)	MT734012	ND	ND	–
Pp no. 32	*Lysinibacillus* sp. (99.33)	MT734013	–	–	+
Pp no. 33	Staphylococcus saprophyticus (99.61)	MT734014	ND	ND	+
Pp no. 34	Lysinibacillus fusiformis (99.13)	MT734015	ND	–	+
Pp no. 35	*L. fusiformis* (99.57)	MT734016	–	–	+
Pp no. 36	Bacillus wiedmannii/*B. proteolyticus* (96.31)	MT734017	ND	–	+
Pp no. 37	*L. fusiformis* (99.31)	MT734018	–	–	–
Pp no. 38	*S. saprophyticus* (99.13)	MT734019	ND	–	–
Pp no. 39	*S. saprophyticus* (99.31)	MT734020	ND	+	+

a + and – indicate presence and absence, respectively; ND, not determined; NA, not available; AF, antifungal activity; AB, antibacterial activity; BS, biosurfactant production.

b The numbers in parenthesis indicate the percent similarity to the reported species.

### Location of the microbes on *P. polytes* wings.

**FIG 1:**
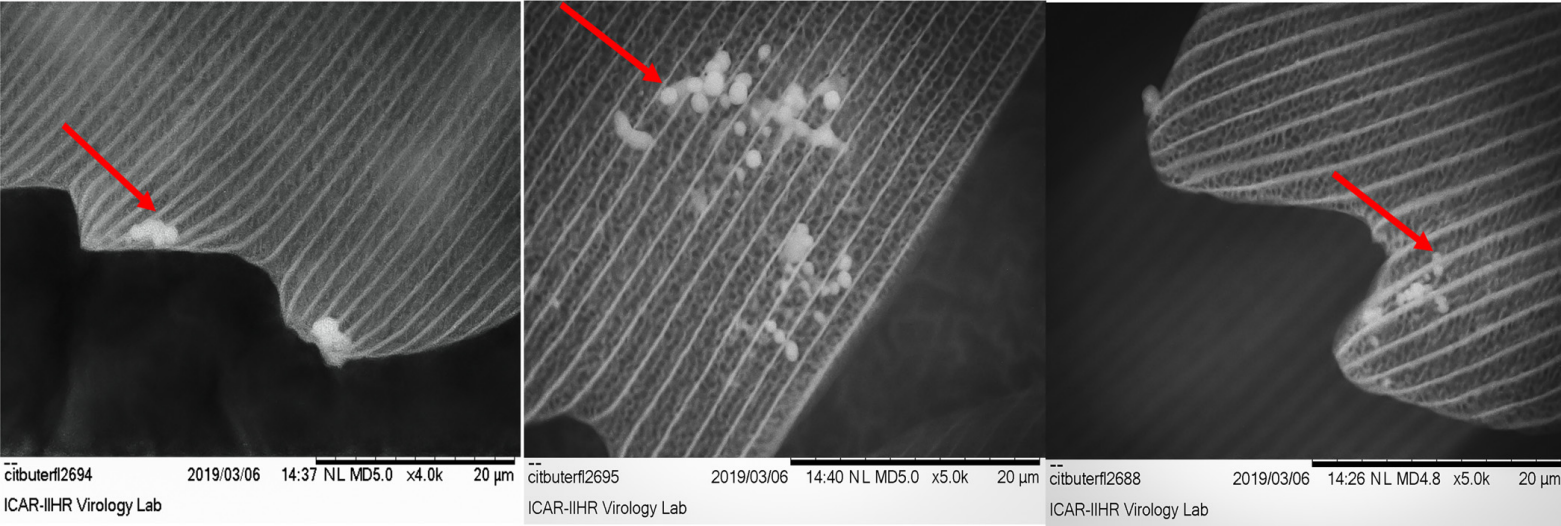
Scanning electron microscopy (Hitachi Tabletop TM3030) was performed on unprocessed citrus butterfly (*Papilio polytes*) wings clipped under sterile conditions from 12- to 1- h-old emerged adults that had been maintained aseptically. The arrows indicate the microbes present on the scales of the wing.

To determine the location of these microbes on the fragile butterfly wings, scanning electron microscopy (SEM) studies were carried out on unprocessed wings. The SEM studies revealed sparsely placed spherical and ovoid- to rod-shaped bacteria under the scales, arranged in layers on the butterfly wings (Fig. 1). The microbes were present beneath the scales and were hard to locate sometimes.

### Biosurfactant properties of wing isolates.

A large number of microbes, bacteria in particular, are known to produce biologically active surface compounds (18). Wing-associated bacteria with such a property could potentially change the hydrophobicity or hydrophilicity of the wing surface. The bacterial isolates were therefore tested for biosurfactant production that might regulate wing surface properties, using the oil spread and the flat drop methods. Surprisingly, a total of 25 isolates tested positive for biosurfactant production in at least one of the two tested methods. A total of four isolates, *B. zhangzhouensis*/*B. pumilus* (Pp no. 5, 25, and 27) and *B. safensis*/*B. pumilus* (Pp no. 18) tested positive for biosurfactant production in both the methods tried, as shown in Table 2.

**TABLE 2:** Results of biosurfactant production assays of the isolates^*a*^

Isolate no.	Oil spread method	Flat drop method
Pp no. 1	+	–
Pp no. 2	+	–
Pp no. 3	–	–
Pp no. 4	–	–
Pp no. 5	+	+
Pp no. 6	–	+
Pp no. 7	–	+
Pp no. 8	+	–
Pp no. 9	–	–
Pp no. 10	–	–
Pp no. 11	+	–
Pp no. 12	+	–
Pp no. 13	+	–
Pp no. 15	+	–
Pp no. 16	–	–
Pp no. 17	–	+
Pp no. 18	+	+
Pp no. 19	+	–
Pp no. 20	–	–
Pp no. 21	–	–
Pp no. 22	–	–
Pp no. 23	–	–
Pp no. 24	+	–
Pp no. 25	+	+
Pp no. 26	–	–
Pp no. 27	+	+
Pp no. 28	–	+
Pp no. 29	+	–
Pp no. 30	–	+
Pp no. 31	–	–
Pp no. 32	+	–
Pp no. 33	+	–
Pp no. 34	+	–
Pp no. 35	+	–
Pp no. 36	+	–
Pp no. 37	–	–
Pp no. 38	–	–
Pp no. 39	+	–

a + and –, presence and absence, respectively, of biosurfactant.

### Antibacterial potential of wing isolates.

Butterfly wings are mostly made of cuticle and are provided structure and nourishment by the hemolymph channeling through the network of veins present on the wings. The discovery of microbes on the wing surface of butterflies was surprising since the wings are not nutrient rich. We therefore tested the biosurfactant production capability and antimicrobial activity of these microbes in bioassays. Antibacterial assays using known entomopathogenic bacterial strains Pseudomonas fluorescenc (19, 20), Bacillus subtilis (21), and Bacillus amyloliquefaciens (22, 23) showed that the tested isolates from the wing displayed mild to very strong antibacterial properties. Isolates Pp no. 7, 27, and 28 displayed mild to very strong antibacterial effects on all three entomopathogenic bacteria tested, as shown in Table 3.

**TABLE 3:** Antibacterial assays performed on known entomopathogens using the citrus butterfly wing microbial isolates

Isolate no.	Anti-bacterial assay against^*a*^		
	*P. fluorescence*	B. subtilis	*B. amyloliquefaciens*
Pp no. 1	–	+	–
Pp no. 5	–	++	+
Pp no. 6	–	++	++
Pp no. 7	+++	++	+
Pp no. 12	–	–	+
Pp no. 17	+++	–	–
Pp no. 18	–	–	++
Pp no. 22	+++	++	–
Pp no. 25	–	++	++
Pp no. 27	+++	++	+
Pp no. 28	+++	++	+
Pp no. 29	+++	–	++
Pp no. 30	–	++	+
Pp no. 32	–	–	+
Pp no. 35	–	–	+
Pp no. 36	–	–	+

a +, mild; ++, strong; +++, very strong; –, noninhibiting.

### Antifungal potential of wing isolates.

Cuticles can be a source for fungal growth in insects such as butterflies that move around freely in the environment, exposed to fungal spores which could readily germinate on the wings and cause pathogenicity. The possibility of an antifungal role of the wing isolates was therefore explored in assays on two known entomopathogenic fungi, namely, Beauveria bassiana and Metarhizium anisopliae. The results showed that the wing isolates displayed mild to very strong antifungal properties toward the entomopathogenic fungi tested. Isolates Pp no. 6, 7, 17, 18, 25, 27, and 28 displayed mild to very strong antifungal effects on B. bassiana and *M. anisopliae*, as shown in Table 4.

**TABLE 4:** Antifungal assays performed on the known entomo-pathogenic fungi using the citrus butterfly wing microbial isolate

Isolate no.	Antifungal assay against^*a*^			
	Beauveria bassiana		Metarhizium anisopliae	
	Growth (cm)	Inhibition zone (cm)	Growth (cm)	Inhibition zone (cm)
Pp no. 1	NI		NI	
Pp no. 2	NI		NI	
Pp no. 6	2.13	3.10	3.75	4.63
Pp no. 7	2.70	4.00	4.50	5.83
Pp no. 9	NI		NI	
Pp no. 11	NI		NI	
Pp no. 17	3.73	4.33	3.73	4.88
Pp no. 18	2.23	2.68	2.63	3.93
Pp no. 25	2.50	3.93	1.88	4.23
Pp no. 27	1.90	2.28	1.90	2.28
Pp no. 28	1.95	2.75	3.05	4.13
Pp no. 35	NI		NI	
Pp no. 37	NI		NI	

a NI, noninhibiting.

### Entomopathogenic effects of *B. safensis*.

**FIG 2:**
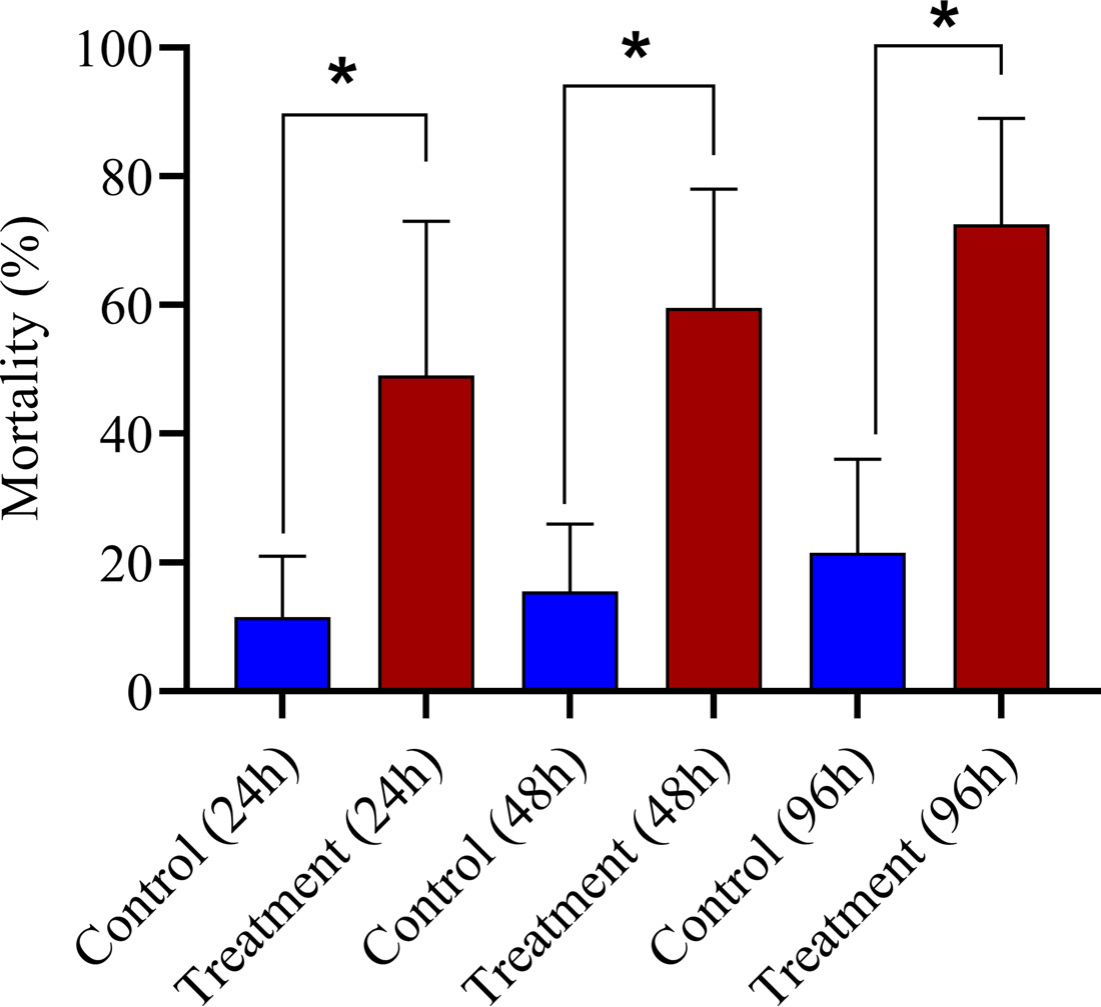
Entomopathogenic property of *B. safensis* against late instar nymphs of mealybugs, *Planococcus citri*. Mortality (%) of mealybugs at time intervals of 24 h, 48 h, and 96 h (96h) after topical application with bacterial isolate Pp no. 7 (*B. safensis/pumilus*). The control bars indicate the mortality of mealybugs (%) that were just treated with nutrient broth. The four asterisks indicate significant data.

Since the bacterial isolates displayed the potential for biosurfactant production and also antimicrobial effects, they were tested for entomopathogenic effects on the notorious pest mealybugs, which have a waxy coating on their body that prevents penetration of insecticide/chemical sprays. As a bioprospecting feature, one of the bacterial isolates, Pp no. 7 (*B. safensis/B. pumilus*), which exhibited robust antibacterial and antifungal properties, was therefore chosen to be tested for its entomopathogenicity against a highly polyphagous citrus mealybug (Planococcus citri), a serious pest of several crop plants. In this study, significant mortality of mealybugs (*P < *0.0001) was observed in detached leaf assays at 24, 48, and 96 h (Fig. 2). The results were on par with a certain nonphytotoxic chemical composition that is used in sprays to kill mealybugs (Kamala Jayanthi and Sravan Kumar, unpublished results).

### Phylogenetic analysis of the wing isolates.

**FIG 3:**
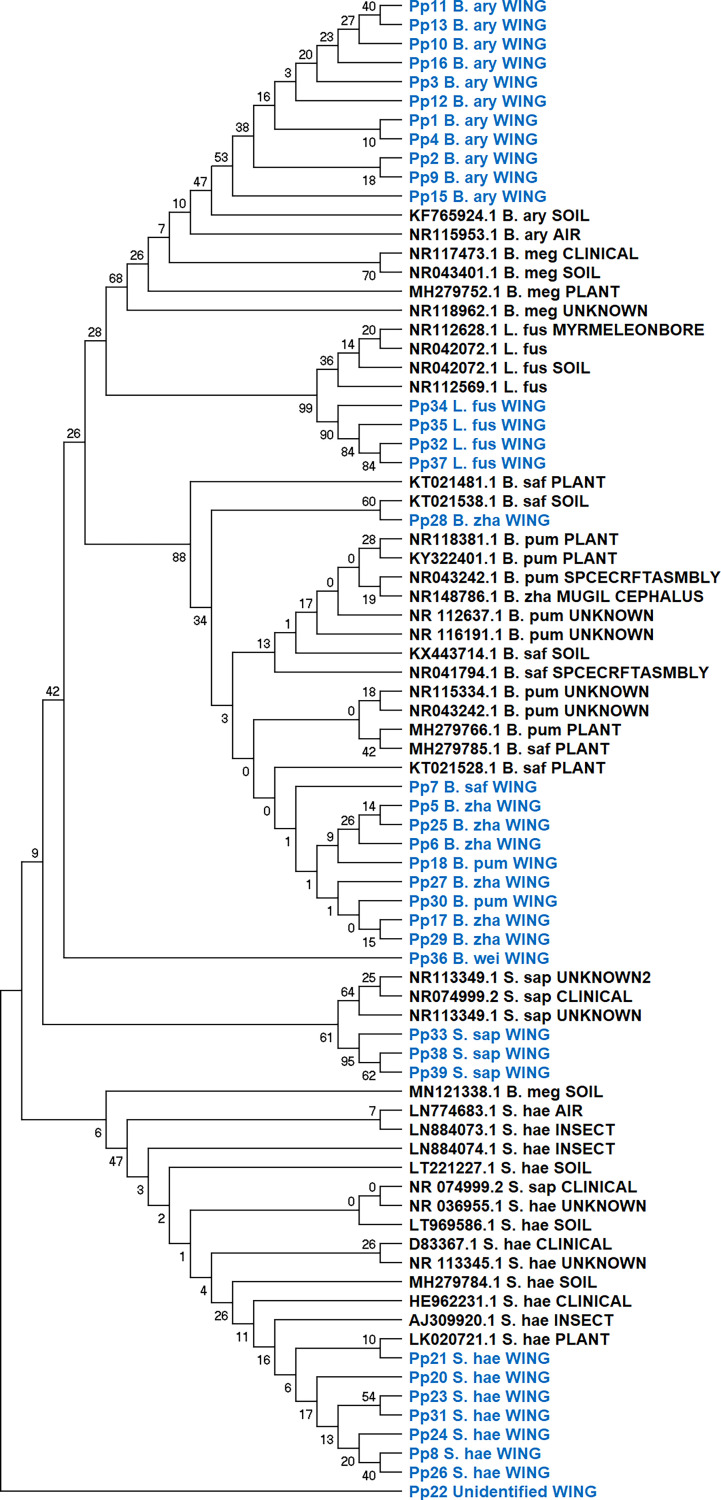
Phylogenetic analysis of the wing isolates with same species isolates (other sources) from NCBI. The maximum likelihood tree is shown for the 16S rRNA gene. “WING” depicts the wing isolates, and the other isolation sources are represented. Bootstrap values are shown on the branches. The wing isolates are labeled in green, while the NCBI-obtained sequences are labeled in black. Support values are provided in the figure.

The isolates in this study have been reported to have diverse characteristics, but their occurrence on the butterfly wing is quite perplexing. The possibility of their origin and association was investigated with a phylogenetic analysis of the isolates with same bacterial species isolated from different sources using sequences available at NCBI. None of the isolates showed evidence of a random occurrence; the specific clades or individuals always associated closely with a particular sampling source namely, rhizosphere, insect source, or plant source from which they were isolated. All the wing isolates were seen to cluster together consistently with the specific species, and based on the available sequences, they could not be separated. One isolate of *B. zhangzouensis* (Pp no. 28) did not cluster with the same species, suggesting they are distinct strains. Since the complete 16S rRNA gene sequence was not available for all isolates to bring out the specific differences, some of the hits in the NCBI database during identification could be the closest similarity hits (Fig. 3).

## DISCUSSION

Microbes are known to exist in association with animal as well as plant systems. They influence various features in the associated hosts, such as digestion, reproduction, immune system, etc. (24, 25). Bacterial communities are known to exist on the external surface of an organism as commensals and provide protection such as in attine ants (26). Recent reports show the occurrence of adult-associated microbiomes in the guts of *Heliconius* butterflies, which show some stable populations among conspecifics. Though they have varied characteristics, their importance to the association with the insect seems unclear (27). In a first of its kind study, we have found that the wings of citrus butterfly *P. polytes* harbor bacteria with distinct characteristics, reflecting on an unexplored microbe world under the tiny niches formed by the arrangement of microscales on their wings.

Considering that microbes associated with the wing are a transient population, some functional aspects of these microbes were tweaked to find a correlation between their occurrence on the wings and their utilities. Most bacterial isolates recovered from the butterfly wings were *Bacillus* species, some of which have interesting features based on previous findings. Bacillus megaterium, identified in our study, is known to exist in diverse habitats and has several industrially competent properties that have enabled its use for industry-grade protein production (28). One of the most intriguing *Bacillus* spp. identified among the isolates is *B. safensis*, a close relative of Bacillus pumilus. First isolated from a space craft assembly unit, *B. safensis* is a known endophyte with plant growth-promoting properties (29), and its hydrocarbon degrading ability (30) has seen use in phytoremediation of oil-contaminated soil (31). Recent reports have suggested its ability to closely associate with human-pathogenic fungi and break down their pathogenicity by inhibiting virulence factors (32). More recently, reported species of *Bacillus*, Bacillus zhangzhouensis, which has the ability to promote plant growth in colder habitats (33, 34), was also identified among the isolates. Lysinibacillus fusiformis, an isolate identified from the butterfly wings, is a known endophyte with plant growth-promoting (35, 36) and biosurfactant-producing properties (37). Here, presence of plant-associated bacteria on butterfly wings just highlights the possibility that the microbes could have come into a chance association with the insect through its diet and habitat dependency on plants. Occasional identification of Staphylococcus strains which are potential human-pathogenic bacteria is not unusual, since the existence of human pathogens and their transmission has been reported in several insects (38, 39). The phylogenetic analysis clusters them with soil-isolated species.

Previous studies have shown that butterfly wings are among the most hydrophobic surfaces in nature (40). Our study illustrates that the bacterial isolates 5 (Bacillus zhangzhouensis/Bacillus pumilus), 18 (Bacillus pumilus), 25 and 27 (*B. zhangzhouensis*/*B. pumilus*) identified from the butterfly wings are capable of producing biosurfactants which are antagonistic to the hydrophobic nature of the wing surface but could contribute to other ecological effects such as antimicrobial effects. Generally, the *Bacillus* species are known to produce a broad spectrum of lipopeptide biosurfactants (41, 42), and certain species such as *B. safensis* are efficient producers of biosurfactants (43). Assessment of historic evidence for beneficial microbes present on insect exoskeletons, such as on attine ants, suggests that symbiotic *Actinobacteria* growing on their cuticle actually produce antimicrobial compounds that serve a protective function against other pathogens (26). In speculation, the antibacterial properties displayed by some of the wing isolates in this study and also reported earlier (44, 45) might suggest a similar role in butterflies; while more in-depth studies will need to be carried out to prove this aspect, the identification of these microbes forms the first step toward such understanding. Additionally, the isolates (Bacillus safensis/*B. pumilus*, *B. zangzhougeus*/*B. pumilus*) were capable of growth inhibition in the known entomopathogens tested, suggesting that these isolates might have a possible biotechnological application. Whether they provide any mutual benefit to the host in this case is an open question. Antifungal properties of biosurfactants produced by *Bacillus* species have been reported earlier (46), and similar antifungal properties were observed in the wing isolates of *Bacillus* spp. in the present study.

A perplexing aspect of this wing-bacterium association is nutrient availability, and considering that most *Bacillus* spp. are known to survive under extremely low nutrient conditions (47), the wing surface might provide just enough for the microbes to survive. Previous studies have identified the ability of B. megaterium to produce extracellular amylases (48), which might possibly help it and other bacteria survive on the meconium that oozes out of the wing veins when the hatched butterflies spread their wings (49, 50). With regard to the origin or source of the microbes, there is a likelihood that the insect acquired them from its gut during metamorphosis. Incidentally, butterfly larvae have been reported to represent the bacterial communities of their diet, and the transition from larval to pupal stage typically does not influence the microbiome of the gut (51), but the composition and structure of bacterial communities simplify during metamorphosis (52). If the wing microbes are acquired during metamorphosis and consecutive emergence, identification of fewer bacterial species on the wings is suggestive of voiding-associated loss of bacterial communities from the larval gut during pupa formation and metamorphosis into the adult form (53). The degradation products and residual nutrients are excreted in the form of meconium postemergence. Phylogenetic analysis of the wing isolates with other isolates of same species showed close clustering of the wing isolates, and the clades were associated with mostly plant-, soil-, or insect-dwelling bacteria. Incidentally bacterial isolates that were identified from wings in the present study have earlier been reported as soil-dwelling or plant-associated, suggesting that the meconium could possibly be the source of these bacteria. The nonavailability of the entire 16S rRNA sequence for the isolates limits interpretation since the structural aspects cannot be taken into account for a better phylogenetic understanding of these isolates. A recent review of bacterial symbionts of *Lepidoptera* (54) highlights the occurrence of very similar bacterial species associated with lepidopterans, including the Staphylococcus spp. Studies of Manduca sexta caterpillars also highlight the occurrence of a nonstable population of microbes in the guts of lepidopterans which do not form a stable symbiotic association. The microbial populations seem to differ among individuals in a population collected from the same place (55). Here, we were able to show the occurrence of microbes having specialty features on citrus butterfly wings, which provides such a potentially transient microbial population a safe niche to thrive. Whether these microbes are just hitchhikers or are potentially symbiotic bacteria acquired through ingestion can only be speculated for now. Explorative studies involving wild-collected and laboratory-reared insects and analysis of their microbial populations will help us understand the possibility of an ectosymbiotic relationship better.

### Conclusions.

The findings reported here for the first time showcase the presence of multifaceted bacteria on the wings of the citrus butterfly *Papilio polytes*. Many of the bacterial species identified had functional properties with potential benefits to the host and might be useful in controlling important agricultural pests such as mealybugs. The study highlights very interesting unresolved issues, such as how butterflies acquire microbes on the exterior surfaces of their wings and the route taken by these microbes to associate with them. Microbial communities associated with butterfly species living in diverse habitats and environments need to be explored and studied to understand such mutual relationships. The diverse characteristics of the wing microbes do seem to suggest that they are a transient population which might change with a change in habitat or environment of the butterfly. Deciphering the transmission path of the observed microbes on the butterfly wings and whether the magnitude of their associated benefits is actually true are important aspects to probe. The findings here open up a novel field of research on the ectosymbiotic association between insects and microbes. Further research is being undertaken to explore the above-described aspects.

## MATERIALS AND METHODS

### Butterfly collection and microbe isolation.

The pupae of common mormon, *Papilio polytes* L. (a swallowtail citrus butterfly distributed across Asia), maintained on citrus plants were collected from (National Centre of Biological Sciences, Bangalore, India). The pupae were surface-cleaned with 1% bleach followed by 70% alcohol and rinsed with sterile distilled water to get rid of the contaminants on the surface which could have arisen during handling and transfer. The disinfected pupae were transferred aseptically to sterile containers and allowed to eclose at room temperature (RT, 27 ± 2^o^C) in aseptically maintained laminar flow chambers to prevent contamination. Then, 12 to 15 h posteclosion, the containers were opened aseptically, and the butterfly was put to sleep using a swab of ethyl acetate. A pair of hind and fore wings from each butterfly was clipped using sterile scissors and patched onto nutrient agar (NA) plates for 30 sec. A total of eight butterflies were used in two biological replicates (two sets of four insects each procured at different times). Both the dorsal and ventral sides were patched. The NA plates were incubated at RT for 24 h in sterile chambers, and the colonies obtained were streak-purified. Single isolated colonies were further used for cultures.

### Microbe culture and identification.

Individual colonies of different morphologies and colors were picked up and streak-purified on nutrient agar and brain heart infusion agar plates. A single isolated colony from each of the streaks was inoculated in 2 mL nutrient broth/brain heart infusion broth and allowed to grow at RT for 24 h. The cultures were then spun down, and an aliquot of each was used to make glycerol stock, while the rest was used for genomic DNA extraction. Genomic DNA was extracted from the cultures using a Genetix Nuclepore DNA extraction kit following the manufacturer’s protocol.

### 16S rRNA PCR and sequencing.

The universal 16S rRNA primers 8F (AGAGTTTGATCCTGGCTCAG) and 1492R (AAGTCGTAACAAGGTARCCGTA) were used to amplify the 16S rRNA gene DNA fragment with the following PCR conditions: initial denaturation at 94°C for 5 min followed by 30 cycles of denaturation at 94°C for 1 min, annealing at 57°C for 30 sec, and an extension time of 30 sec at 72°C. A 2× ready master PCR mix (New England Biolabs [NEB]) was used for the reaction. The amplified products were confirmed by gel electrophoresis on a 1% agarose gel, column-purified, and sequenced. Sequences obtained were submitted to a BLAST search using the NCBI nucleotide BLAST database tool (https://blast.ncbi.nlm.nih.gov/Blast.cgi), and the best matches with 95% or more identity were considered. The identified isolates were then tabulated (Table 1) along with the accession numbers.

### Scanning electron microscopy of butterfly wings.

Scanning electron microscopy (SEM) was used to observe the dorsal and ventral surfaces of the butterfly wing for presence of microbes. The unprocessed pair of hind and fore wings was clipped and glued onto copper pin mounts with a double-sided carbon adhesive tape, and SEM imaging was done using a scanning electron microscope (Hitachi Tabletop TM3030; from the Department of Plant Pathology, Indian Institute of Horticultural Research, Bangalore, India). Any microbes observed were imaged.

### Biosurfactant detection assays.

Biosurfactant assays were carried out using two different methods, the drop collapse test (56) and the oil spreading method (57). Cultures (24 h old) of isolates were spun down, and the supernatant was collected in separate tubes. In an enzyme-linked immunosorbent assay (ELISA) plate, 100 μL of crude oil was added to each of the wells and allowed to stabilize for 5 to 6 h. Then, 200 μL of each of the test supernatant isolates was added in separate wells along with a negative control (Milli-Q water) and a positive control (0.01% sodium dodecyl sulphate [SDS]) solution in water and tested for the appearance of the supernatant drop. A flat drop indicated the presence of surfactant activity, while a spherical drop indicated its absence. The results were recorded as the presence (+) and absence (–) of surfactant activity. In the oil spreading method, 40 mL of sterile Milli-Q water was added to a petri plate, and then a few drops of crude oil were added on the surface. About 100 μL of the supernatant was then slowly added over this. The culture was marked positive for the presence of surfactant if the oil spread to the periphery and diffused, and it was marked negative for the absence of surfactant if there was no spread of oil. The results obtained were tabulated (Table 2).

### Antibacterial assays.

Antibacterial activity was tested in bioassays using the three entomopathogenic bacterial strains, Pseudomonas fluorescence, Bacillus subtilis, and Bacillus amyloliquefaciens. The agar well diffusion method was used for this test (58), where 200 μL of an overnight culture of each of the three bacterial strains was plated on NA and allowed to grow for 12 to 14 h. A bacterial lawn was observed, and four plugs were then cut out of the culture plates at the center of the four quadrants. Then, 100 μL of overnight culture of the wing isolates was then added to the wells by cutting out the medium plugs in all four quadrants. The zone of clearance (starting from the edge of the well to end of the clear zone with no growth) was quantified after 48 h as very strong (+++), strong (++), mild (+), and noninhibiting (–) based on the thickness of the clear zone surrounding the well in the plate (Table 3).

### Antifungal assays.

Potato dextrose agar (PDA) plates were plated with a 24-h culture of the isolate to be tested, and a small plug of the entomopathogenic fungal mycelia (Beauveria bassiana and Metarhizium *anisopliae*) was placed at the center of the plate. The growth zone of the fungal plug was observed for 48 h, and measurements of growth area and the area of clearance were recorded. The zone of inhibition was calculated using the formula (zone of clearance – zone of growth = zone of inhibition), inhibition was quantified based on the thickness of the clear zone (cm) surrounding the fungal plug in the plate, and the extent to which the fungus grew was recorded. The results obtained were tabulated (Table 4).

### Entomopathogenic assays.

A few isolates exhibited antibacterial and antifungal properties along with biosurfactant production properties. Considering that such features could be useful in biocontrol management, one isolate that exhibited robust antibacterial and antifungal properties was selected to test as a biocontrol agent on mealybugs that produce a wax coating making them less responsive to insecticides and other control methods. Bioassays were done on the highly polyphagous citrus mealy bug (*Planococcus citri* [Risso]) using a detached leaf assay. Ten late instars of *P. citri* nymphs were put on freshly plucked Annona squamosa leaves (which had moist tissue paper underneath that served as a base) in petri plates under aseptic conditions. An overnight culture of the specific isolate was used as the test, and nutrient broth was used as the control. Ten replicates of such plates were used for each experiment, and the experiment was repeated three times. The percent mortality was recorded for each plate at 0, 24, 48, and 96 h posttreatment. The data were subjected to analysis of variance (ANOVA).

### Evolutionary analysis by the maximum likelihood method.

Representative sequences of the same16S rRNA were downloaded from a public database https://www.ncbi.nlm.nih.gov/ for all the species identified in this report, but with different isolation sources. The sequences were aligned with those from the wing isolates using MUSCLE (59), and DNA alignment was done using MEGA X (60). The aligned sequences were then used for phylogenetic analysis. The evolutionary history was inferred using the maximum likelihood method and Tamura-Nei model (61). The bootstrap consensus tree inferred from 500 replicates was taken to represent the evolutionary history of the taxa analyzed (62). Branches corresponding to partitions reproduced in less than 50% of bootstrap replicates were collapsed. The percentage of replicate trees in which the associated taxa clustered together in the bootstrap test (500 replicates) is shown next to the branches (62). Initial trees for the heuristic search were obtained automatically by applying the neighbor-join and BioNJ algorithms to a matrix of pairwise distances estimated using the maximum composite likelihood (MCL) approach and then selecting the topology with the superior log likelihood value. This analysis involved 79 nucleotide sequences. There was a total of 3,156 positions in the final data set. Evolutionary analyses were conducted in MEGA X (60). The NUWICK tree was then exported to FigTree (version 1.4.4) for figure preparation.

### Data availability.

The 16S rRNA sequences have been deposited with GenBank and are available under accession no from MT733985 to MT734020 (Table 1).
